# Understanding the Role of Genetic Testing in Diagnosing a Complex Pediatric Case

**DOI:** 10.1002/ccr3.72899

**Published:** 2026-06-09

**Authors:** Giavanna Verdi, Aleksandra Foksinska, Elizabeth L. Nichols, Erinn O. Schmit, Andrew Watson, Madeline Eckenrode, Nathaniel H. Robin

**Affiliations:** ^1^ Department of Genetics Heersink University of Alabama at Birmingham School of Medicine Birmingham Alabama USA; ^2^ Department of Pediatrics Heersink University of Alabama at Birmingham School of Medicine Birmingham Alabama USA; ^3^ Hugh Kaul Precision Medicine Institute University of Alabama at Birmingham Birmingham Alabama USA

**Keywords:** alopecia, clinical genetics, exome sequencing, kidney, missense variant, recurrent infections, variant of uncertain significance, ZPR1

## Abstract

We report the case of a 13‐month‐old female with multiple congenital anomalies including microcephaly, generalized hypotonia, sensorineural hearing loss, visual impairment, alopecia, and hypoplastic kidneys with chronic kidney disease, and dysmorphic craniofacial features. Extensive metabolic and immunodeficiency workups were unremarkable. Genetic evaluation was conducted in a stepwise approach, and with exome sequencing (ES), we identified two compound heterozygous variants of uncertain significance in the ZPR1 gene. These variants resembled previously reported pathogenic ZPR1 variants associated with a syndrome characterized by growth restriction, craniofacial abnormalities, alopecia, and hypoplastic kidneys. With variant interpretations and clinical comparisons to our patient, we strongly suspect a likely pathogenic role for the ZPR1 variants in this patient's multisystem phenotype, highlighting the importance of exome sequencing in diagnosing medically complex pediatric patients.

## Key Clinical Message

There is diagnostic utility of exome sequencing in pediatric patients with multiple congenital anomalies and/or evolving phenotypes. The early incorporation of comprehensive molecular genetic testing can enhance diagnostic yield, as well as prevent unnecessary tests, reduce hospitalization, and tailor patient care more effectively.

## Graphical abstract

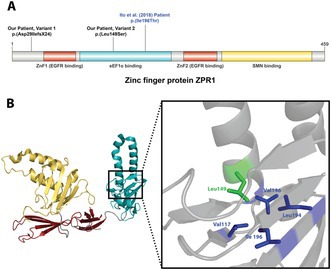

## 1 Introduction

Genomic testing has become an integral component of the diagnostic evaluation for individuals presenting with multiple congenital anomalies. Exome sequencing (ES) is particularly beneficial when the clinical phenotype is nonspecific or evolves over time, providing diagnostic clarity when targeted testing is nondiagnostic. In recent years, broad sequencing approaches, including ES and genome sequencing, have significantly enhanced the identification of underlying genetic etiologies in complex clinical presentations [1]. Here we present a case in which ES identified the underlying etiology in a patient with a complex clinical presentation.

## 2 Case History and Examination

**FIGURE 1:**
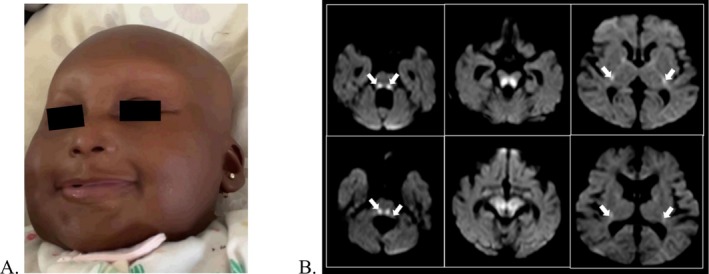
Investigations of our patient. (A) Clinical photograph of the patient in this report, demonstrating distinctive craniofacial features. Consent for publication was obtained from the patient's legal guardian at time of manuscript production. (B) MRI Brain demonstrating T2 hyperintensity along the bilateral dorsal pons and midbrain, cerebellar peduncles, and internal capsules, corresponding with concerns for diffusion restriction. These findings can be suggestive of a metabolic derangement affecting deep white matter tracts and brainstem structures.

We present a 13‐month‐old female patient born at 36.6 weeks gestational age with microcephaly, generalized hypotonia, sensorineural hearing loss, visual impairment, alopecia, hypoplastic kidneys, chronic kidney disease with associated electrolyte abnormalities, elevated transaminases, global developmental delay, and feeding intolerance with chronic severe malnutrition ultimately requiring gastrojejunostomy tube placement. Other complications included stress‐related hyperglycemia, elevated hemoglobin A1c, and glucosuria; multiple viral infections leading to rapid‐onset decompensated shock; and normocytic anemia. Distinctive craniofacial features were notable for microcephaly, narrowed temporal regions without evidence of craniosynostosis, deep‐set eyes with hypotelorism, depressed and widened nasal bridge, smooth philtrum, and diffuse alopecia (Figure 1A). Brain MRI demonstrated T2 hyperintensity along the bilateral dorsal pons and midbrain, cerebellar peduncles, and internal capsules, suggestive of metabolic derangement (Figure 1B).

## 3 Investigations

Given these abnormalities along with recurrent hospital admissions for decompensated shock and hyperglycemia in the setting of viral infections, there was concern for an underlying inborn error of metabolism and/or inherited immunodeficiency. Extensive metabolic and immunologic testing, including immunoglobulin levels, plasma amino acids, urine organic acids, and a plasma acylcarnitine profile, was nondiagnostic.

Based on her complex but nonspecific clinical presentation, broad‐based genetic testing was ordered, including a karyotype and chromosomal microarray (CMA). When these were nondiagnostic, ES was performed. Maternal DNA was submitted for targeted sequencing of identified variants, whereas paternal DNA was unavailable at the time of analysis. ES identified several variants; however, the two variants in the ZPR1 gene were most relevant to the patient's clinical presentation.

## 4 Conclusion and Results

The CUBN gene encodes for cubilin, responsible for intrinsic factor–mediated absorption of vitamin B_12_. Pathogenic variants in this gene may lead to vitamin B_12_ deficiencies as well as benign proteinuria [2, 3]. The patient's vitamin B_12_ level was normal at 937 pg/mL, with low homocysteine (< 3 μmol/L) and mildly elevated methylmalonic acid (532 μmol/L). The two CUBN variants, classified as variants of uncertain significance, were considered of no immediate clinical relevance.

The ZPR1 gene encodes a zinc finger protein that acts as a signaling molecule mediating proliferative growth signals. Pathogenic variants have been reported in a syndrome characterized by growth restriction, craniofacial abnormalities, alopecia, and hypoplastic kidneys [4]. Our patient phenotypically resembles previously described cases (Table 1B).

**TABLE 1:** Comparison of genotype (A) and clinical features (B) between our patient's *ZPR1* variants to previously reported individuals with identified *ZPR1* variants [4].

A			
	This study	This study	Ito et al. [4] variant
	Patient variant 1	Patient variant 2	
Variant NM_003904.4	c.84del (p.Asp29IlefsX24)	c.446T>C (p.Leu149Ser)	c.587T>C (p.Ile196Thr)
Variant type	Frameshift	Missense	Missense
Inheritance	Maternal, Heterozygous	Unknown, Heterozygous	Homozygous
Protein domain	N‐terminal region	eEF1α binding domain	eEF1α binding domain
In silico prediction	Likely deleterious (null)	Likely deleterious	Experimentally confirmed deleterious
Predicted functional impact	Complete loss of function	Predicted protein instability/degradation	Confirmed protein instability/degradation

B		
	This study patient	Ito et al. [4] patients
Alopecia at birth	✓	✓ (all four patients)
Hypoplastic kidneys	✓	✓ (all four patients)
Congenital sensorineural hearing loss	✓	✓ (all four patients)
Cleft soft palate		✓ (one of the four patients)
Congenital hip dislocation		✓ (all four patients)
Deep‐set eyes	✓	✓ (all four patients)

*Note:* Variant coordinates are reported according to the GRCh37 human reference genome. In silico predictions were generated using the Protein Variation Effect Analyzer (PROVEAN) [5] and sequence changes of interest are interpreted according to the American College of Medical Genetics and Genomics (ACMG) guidelines.

**FIGURE 2:**
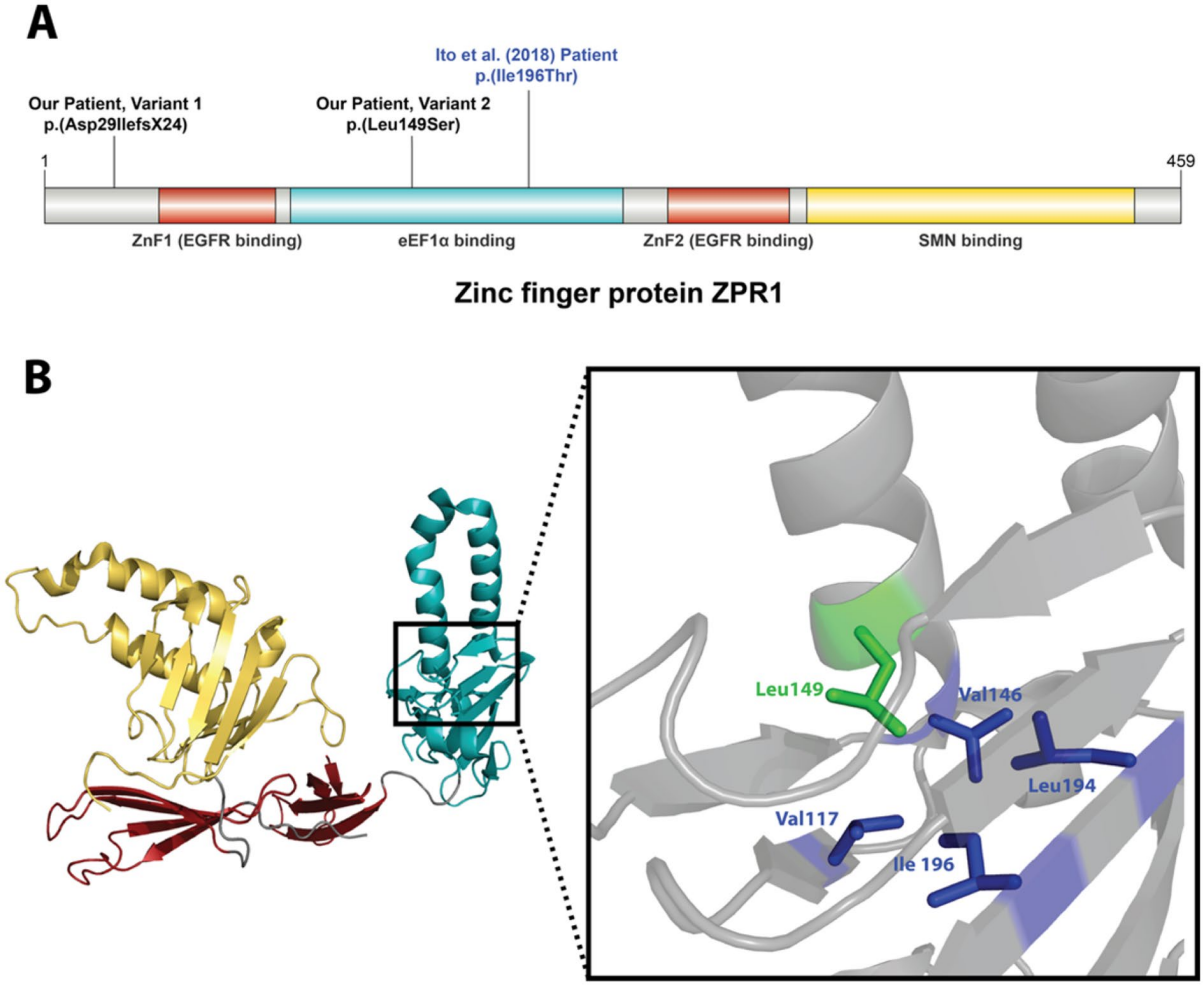
Visualization of ZPR1 protein variants. (A) Schematic diagram of the ZPR1 protein (NP_003895), showing two zinc finger motifs (red), the eEF1α‐binding domain (teal), and the SMN‐binding domain (yellow). The variants identified in our patient are labeled in black, while the previously reported p.Ile196Thr variant from Ito et al. [4] is shown in blue. (B) Structural visualization of the mouse ZPR1 homolog (PDB: 2QKD), with domains colored to match panel A [5]. The inset highlights the location of Leu149 (green) relative to Ile196, which is substituted in the p.Ile196Thr variant. Nearby hydrophobic residues are shown in blue. The mouse and human ZPR1 proteins share 92% identity and 95% similarity across the aligned region (residues 47–440), supporting the use of the mouse structure as a reliable model for human ZPR1. The two‐dimensional protein domains were visualized with IBS 2.0 (Xie et al. [6]), while the three‐dimensional structure was visualized using PyMOL (version 0.99rc6, DeLano Scientific LLC) [7]. A.

Two ZPR1 variants were identified in our patient (Table 1A). The c.84del variant, maternally inherited, results in an early frameshift predicted to produce a null protein lacking all characterized functional domains (Figure 2A). ZPR1 is intolerant to complete loss of function, consistent with embryonic lethality in mouse models [8]. The missense variant p.(Leu149Ser), of unknown inheritance, occurs within the eEF1α binding domain and resembles the previously reported p.Ile196Thr variant [4]. Both involve substitution of hydrophobic residues with polar residues within the same hydrophobic pocket (Figure 2B) and are predicted to destabilize the protein [5, 9].

A report by Quintana et al. [10] expanded the ZPR1‐associated phenotype to include growth hormone resistance and progressive liver fibrosis in addition to alopecia and hypoplastic kidneys. The patient in our study shares key features with those described by Quintana et al., further supporting the likely pathogenic role of these ZPR1 variants and extending the genotype–phenotype correlation (Table 1A, Figure 2).

Currently, phasing of the two variants is unknown, as parental DNA was incomplete. Since we know one of the ZPR1 variants was maternally inherited, these variants are most likely in trans configuration. Additional methods such as long‐read sequencing or allele‐specific PCR could further help determine cis versus trans configuration. While these ZPR1 variants account for most of the observed clinical features, some manifestations may involve additional factors not yet identified.

## 5 Discussion

This case underscores the growing importance of ES in diagnosing complex pediatric presentations when standard diagnostic approaches are inconclusive. Recent evidence‐based guidelines from the American College of Medical Genetics and Genomics (ACMG) advocate for ES or genome sequencing (GS) as a first‐ or second‐tier diagnostic test for children with unexplained congenital anomalies or unrelated clinical features [7]. There is evidence of a higher diagnostic yield and potential cost‐effective medical practice compared with traditional testing methods [1].

Furthermore, systematic reviews of ES and GS applications in pediatric populations have shown that genomic sequencing may not only increase diagnostic accuracy but also frequently impact and alter clinical management [4]. Aligning with these findings, the use of ES in our patient led to a potential diagnosis, ultimately facilitating more targeted care and avoiding unnecessary investigations. This case reinforces the value of incorporating exome sequencing into the early diagnostic workup for children with medically complex clinical presentations.

## 6 Learning Points

1. There is diagnostic utility of exome sequencing (ES) in pediatric patients with multiple congenital anomalies and/or evolving phenotypes.
2. This case illustrates the potential of early incorporation of comprehensive molecular genetic testing to enhance diagnostic yield, as well as prevent unnecessary tests, reduce hospitalizations, and tailor patient care more effectively.
3. The phenotype of those with identified variants at the *ZPR1* gene is further expanded and may suggest a likely pathogenic role.

## Author Contributions

**Giavanna Verdi:** conceptualization, data curation, investigation, writing – original draft, writing – review and editing. **Madeline Eckenrode:** supervision, validation, writing – review and editing. **Aleksandra Foksinska:** data curation, investigation, visualization, writing – review and editing. **Elizabeth L. Nichols:** conceptualization, resources, writing – review and editing. **Erinn O. Schmit:** conceptualization, resources, writing – review and editing. **Nathaniel H. Robin:** supervision, validation, writing – review and editing. **Andrew Watson:** conceptualization, resources, writing – review and editing.

## Funding

The authors have nothing to report.

## Ethics Statement

The authors have nothing to report.

## Consent

Written informed consent was obtained from the patient's legal guardian for publication of this case report and any accompanying images.

## Conflicts of Interest

The authors declare no conflicts of interest.

## Data Availability

The data that support the findings of this study are available on request from the corresponding author. The data are not publicly available due to privacy or ethical restrictions.
